# Gait Characteristics in Patients With Ankylosing Spondylitis: Protocol for a Systematic Review

**DOI:** 10.2196/12470

**Published:** 2019-05-01

**Authors:** Julie Soulard, Nicolas Vuillerme, Jacques Vaillant

**Affiliations:** 1 AGEIS Université Grenoble Alpes Grenoble France; 2 Grenoble Alpes University Hospital Grenoble France; 3 Institut Universitaire de France Paris France

**Keywords:** gait, ankylosing spondylitis, clinical measurements, laboratory measurements

## Abstract

**Background:**

Ankylosing spondylitis is a subtype of inflammatory rheumatic disease, affecting predominantly the axial skeleton and sacroiliac joints. The main clinical manifestations are spinal stiffness and inflammatory back pain, which can potentially affect gait ability of patients with ankylosing spondylitis. However, published studies show discrepancies regarding gait characteristics in ankylosing spondylitis and heterogeneity in terms of task requirement, types of equipment, data collection, and analysis techniques used to assess gait ability of patients with ankylosing spondylitis.

**Objective:**

This review aimed to determine (1) the consequences of ankylosing spondylitis on gait and (2) how gait is assessed in patients with ankylosing spondylitis.

**Methods:**

Three electronic databases—PubMed, Physiotherapy Evidence Database (PEDro), and Cochrane—were searched systematically with no limit on the publication date in order to identify studies satisfying the search criteria. The research focused on original research, using Boolean operators “AND” and “OR” in the combination of the Medical Subject Headings descriptors found in titles or abstracts: (Gait OR Walk OR Walking OR locomotor OR locomotion) AND (ankylosing spondylitis OR spondyloarthritis). Only English-language original articles were included.

**Results:**

As of September 2018, the search was completed, and 168 records were obtained. After screening titles and abstracts, 19 full texts were reviewed. Of those, 17 were included in the review. We are currently in the process of data extraction and synthesis.

**Conclusions:**

The systematic review will provide a synthesis and comprehensive evaluation of published studies on gait characteristics in patients with ankylosing spondylitis. This work is also intended to help identify the likely relevant directions for future research.

**Trial Registration:**

PROSPERO CRD42018102540; https://www.crd.york.ac.uk/prospero/display_record.php?RecordID=102540

**International Registered Report Identifier (IRRID):**

DERR1-10.2196/12470

## Introduction

Ankylosing spondylitis is a subtype of inflammatory rheumatic disease, predominantly affecting the axial skeleton and sacroiliac joints. Ankylosing spondylitis is associated with inflammation or new bone formation, with syndesmophytes and ankyloses visible on radiographs [1]. The main clinical manifestations are spinal stiffness and inflammatory back pain, which yield adverse effects on work ability, work productivity, quality of life, and psychological well-being [2,3]. Gait ability is known to contribute to functional independence and quality of life in patients with stroke [4,5] or following hip arthroplasty [6] and is impaired in patients with ankylosing spondylitis [7]. Indeed, considering the decreased range of movement, pain, and altered posture [8,9] associated with ankylosing spondylitis, previous works have reported that ankylosing spondylitis leads to more cautious gait pattern, shorter stride length, and decreased range of motion at the hip and knee joints [10-12]. However, it is still unclear whether and how gait is modified in patients with ankylosing spondylitis. Previous studies reported that patients with ankylosing spondylitis covered significantly lesser distance than controls during the Six-Minute Walk test [13] and adopted a shorter stride length [12], whereas in other published studies, no statistically significant group differences were reported for the same gait-related parameters [10,11,14]. Furthermore, published studies showed heterogeneity in terms of task requirement, types of equipment, data collection, and analysis techniques used to assess gait ability of patients with ankylosing spondylitis. For instance, gait-related studies encompass an increasingly large variety of tasks, types of equipment, and analysis techniques including, for example, both clinical (eg, Timed-Up-and-Go test and Six-Minute Walk test) and laboratory measurements (kinetic, kinematic, or electromyographic gait analysis), which should be taken into consideration for the assessment of gait ability in patients with ankylosing spondylitis. Thus, this systematic review aims to document the effect of ankylosing spondylitis in gait, specifically focusing on published studies that have reported clinical or laboratory gait measurements in patients with ankylosing spondylitis. More specifically, this review aimed to determine the consequences of ankylosing spondylitis on gait and how gait is assessed in patients with ankylosing spondylitis.

## Methods

This protocol has been registered in PROSPERO (CRD42018102540). We followed the Preferred Reporting Items for Systematic Reviews and Meta-Analysis (PRISMA) statement guidelines provided by Moher et al when conducting our systematic review and meta-analysis [15].

### Inclusion Criteria

Original quantitative and qualitative research studies that assessed gait in patients with ankylosing spondylitis were included. To be eligible for inclusion, studies had to be published in English in peer-reviewed scientific journals.

#### Type of Participants

Studies were included if participants were older than 18 years, with a diagnosis of ankylosing spondylitis.

#### Type of Outcome Measurements

Studies were included if they reported clinical or laboratory gait measurements.

#### Type of Studies

Observational and experimental study designs were included.

### Exclusion Criteria

The following types of studies were ineligible: case reports, abstracts, editorials, conference abstracts, letters to the editor, reviews, and meta-analysis.

We also excluded studies that reported gait outcomes inadequately (without mean and SD, or median associated with interquartile range or first and third quartiles) or those from which it was not possible to extract data from the results section.

### Data Sources and Search Strategy

A computer-aided literature search was conducted in the following electronic databases on June 5, 2018, with no date restrictions: PubMed, Physiotherapy Evidence Database (PEDro), Cochrane library.

Consistent with a similar review, search terms included those related to population, ankylosing spondylitis [16], and the outcome—gait [17]. The search strategy included a combination of the following keywords and Medical Subject Headings terms found in the abstract or title: (“gait” OR “walk” OR “walking” OR “locomotor” OR “locomotion”) AND (“ankylosing spondylitis” OR “spondyloarthritis”).

### Study Selection

Two reviewers independently screened the titles, abstracts, and keywords identified by the search strategy in order to select potentially relevant studies.

After this initial search, full-length texts of the identified potentially relevant studies were obtained. Based on the above mentioned inclusion and exclusion criteria, the two reviewers further screened these full texts to elucidate their eligibility and decide on their inclusion. In case of any disagreement, consensus was reached through discussions between the two reviewers. If no consensus was achieved between the two reviewers, a third reviewer was contacted.

### Risk of Bias in Individual Studies

As our aim is not to evaluate the effect of an intervention, we did not use a risk-of-bias assessment. As mentioned above, our aim was to document the effect of ankylosing spondylitis on gait, specifically focusing on published studies that have reported clinical or laboratory gait measurements in patients with ankylosing spondylitis.

### Data Extraction

**Figure 1:**
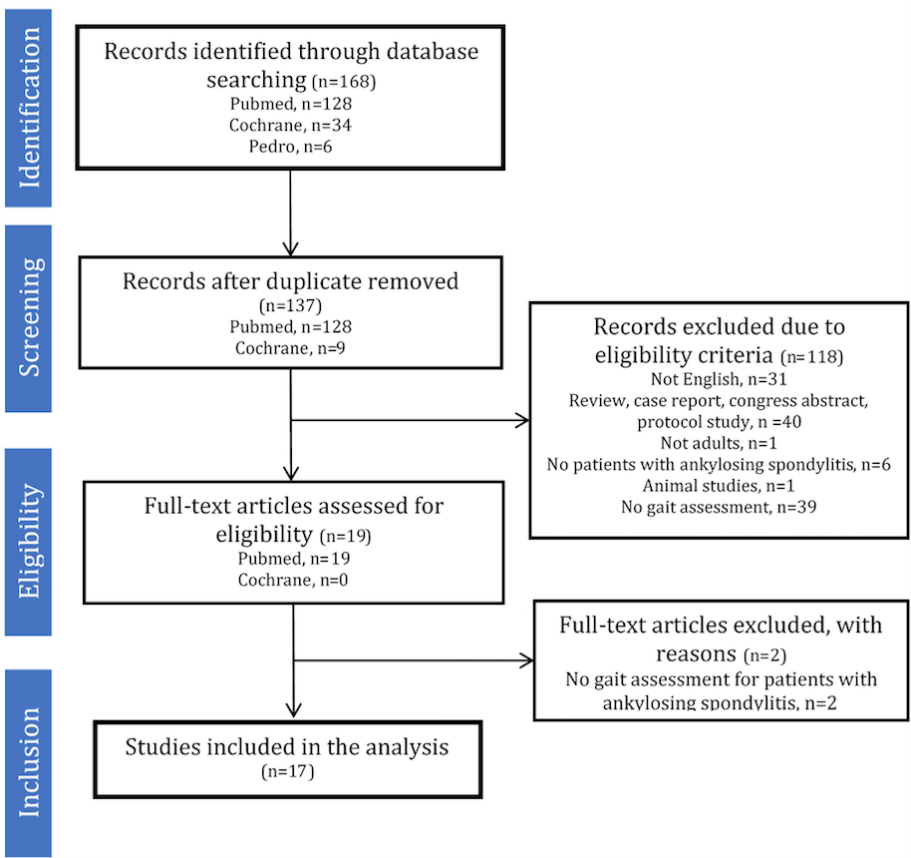
Preferred Reporting Items for Systematic Reviews and Meta-Analysis (PRISMA) flow chart of the selection process.

Following the PRISMA guidelines [15], a flow chart of the selection process was created, with the number of citations reviewed at each stage of the review (Figure 1). Additionally, the following four sets of data will be extracted from the retrieved articles [18]:

- Study characteristics: first author(s), title, year of publication, journal name, and country
- Sample description: sample size, age, gender, weight, height, body mass index, health status, disease duration, functional status measurements, level of pain, description of radiographic damage, biologic medications, Bath ankylosing spondylitis functional index, Bath ankylosing spondylitis disease activity index, and Bath ankylosing spondylitis metrology index
- Methods: task requirement, data acquisition methodology and instrumentation, and parameters assessed
- Main results obtained from gait assessment: clinical measurements of gait (Six-Minute Walk distance and time to complete the Timed-Up-and-Go test) and laboratory measurements of gait such as spatiotemporal parameters (gait speed, stride length, stride time, and cadence) and kinematic parameters (continuous estimate of relative phase, joint range of motion, and joint moments)

Means and SDs or medians associated with interquartile range or the first and third quartiles will be extracted. Two reviewers will independently extract these data from each enrolled study and compare the data for consistency. Any discrepancies between the two reviewers will be resolved at a consensus meeting. If disagreement persists, a third reviewer will be consulted to achieve a final judgment.

## Results

As of September 2018, we have completed the search strategy and obtained 168 records. After screening titles and abstracts, 19 full texts were reviewed. Of those, 17 were included in the review (Figure 1). We are currently in the process of data extraction and synthesis. We expect the final results to be submitted for publication in March 2019.

## Discussion

Considering the importance of gait in functional independence and quality of life, there are a growing number of reviews and reports examining gait characteristics in a wide range of populations [19] with neurological disorders [20-22], hip osteoarthritis [23], diabetes [24], frailty [25], or dementia [26] and in older adults [17]. However, as of September 2018, only one review published in 2015 focused on gait characteristics in rheumatologic patients [7], with only 3 studies reporting results of patients with ankylosing spondylitis [10-12]. Interestingly, the abovementioned review [7] focused on case-control studies only (ie, “studies were included...if they were articles that included a healthy group as means of comparison” [7]). This review did not include studies with clinical measurements of gait, but only studies reporting laboratory measurements (ie, “studies were included...if they reported spatiotemporal, kinematic, kinetic, peak plantar pressure or muscle activity data during gait” [7]). Thus, an update of the published literature is needed.

A strength of this review protocol is that it includes both clinical and laboratory measurements of gait studies on patients with ankylosing spondylitis, reporting precisely the methodology used in each selected study, as recommended by the PRISMA statement (e.g. “how the data was collected and analysed” [15]). Indeed, early identification of gait deficits in patients with ankylosing spondylitis could help us better understand, follow, and predict disease evolution and allow for timely implementation of targeted interventions or treatment to improve gait. Keywords have been chosen based on latest reviews on ankylosing spondylitis [16] and gait [17] separately and were searched in principal databases, assuring the conduct of a systematic review.

However, there are some limitations related to this review that need to be addressed. We assume that the selection and qualitative synthesis of the eligible studies are a subjective process. However, we will seek to minimize this limitation by duplicating our search and having two reviewers conduct the screening process independently [15]. We plan to present the results of this systematic review at international scientific and clinical conferences and publish them in a peer-reviewed scientific journal. The systematic review will provide a synthesis and comprehensive evaluation of published research on gait characteristics in patients with ankylosing spondylitis. Largely, this work is further intended to help identify the likely relevant directions for future research. For instance, from a clinical perspective, we support the idea that an objective and standardized assessment of gait characteristics should be an integral part of every comprehensive assessment of patients with ankylosing spondylitis.

## Abbreviations

PEDro: Physiotherapy Evidence Database
PRISMA: Preferred Reporting Items for Systematic Reviews and Meta-Analysis
